# Effectiveness of Integrating Role Play with Didactic Lecture, Video Review, and Group Discussion in Enhancing Interview Skills of New Psychiatric Nurses

**DOI:** 10.1007/s40596-016-0610-3

**Published:** 2016-10-24

**Authors:** Wen-Ching Chen

**Affiliations:** Yuli Hospital, Ministry of Health and Welfare, Executive Yuan, Taiwan

To the Editor:

In psychiatry, interview skills are considered core abilities [1], especially in managing difficult situations in psychiatric wards. Research has found that poor doctor-patient interviews led to increased errors and noncompliance in medication-taking behavior. Furthermore, psychotic patients, owing to a lack of insight into their own conditions, tend not to comply with demands/requests from psychiatric nurses and refuse to cooperate. Lack of good interview skills among psychiatric nurses is associated with poor treatment adherence, lower patient engagement, and high premature discharge rates [2].

In practice, role play is often integrated with didactic lecture and group discussion: participants review video recordings of role-play and provide feedback to one another. Video feedback is reported to be potentially effective in enhancing the generic interview skills of practice nurses [3].

Twenty nurses working in Yuli Hospital for less than 12 months with no prior interview training were recruited for this quasi-experimental study. It was approved by the Institutional Review Board of Yuli Hospital (YLH-IRB-9903), and the participants signed an informed consent document that clearly stated the study’s purpose, the benefits/risks of engagement, and their right to withdraw at any time. After they were randomly divided into two groups of 10 members each, group 1 and group 2, I used a cross-over design and controlled intervention to compare pre- and post-training performance in role play interviews. A questionnaire solicited demographic data and degree of self-confidence in interviewing patients. At the beginning and end of the study, participants were asked to indicate their agreement (strongly disagree to strongly agree) with sentences describing their self-confidence in six interview skills: opening and closing an interview, using door openers and empathetic statements, stating concrete effects, and determining solutions.

Ten scenarios were generated, each describing a situation encountered regularly in nursing practice, such as patients who are reluctant to engage in activities or who refuse to take medication. Role play interview sessions were conducted in March (interview I), May (interview II), and August (interview III), 2010. In each session, the scenario and the participants’ roles were determined by drawing lots. Each participant served once as an interviewer and once as an interviewee (simulated patient), acting out the situation described. Each interview had to be completed within 5 min and was video-recorded for subsequent review in group discussion sessions.

The interviewer’s performance was scored with an audit tool for desired behavior, which evaluated the six interview skills. A score of 1 was given if the skill was used once, 2 if used twice or more, and 0 if never used. Immediately after each interview, participants entered their scores into a voting machine. Ten scores were collected: one from the interviewee acting as the patient, one from the interviewer self-assessing his/her own performance, and one each from the observing group members. Each participant had seven average scores, each ranging from 0 to 2, for each interview session, including an average score for each of the six interview skills plus an average overall score indicating the participant’s mastery of the interview skill and interview competence in that session. At the end of the study, each participant had three sets of scores for interview I (baseline), interview II, and interview III.

The intervention was the 2-month training program, comprising two didactic lectures followed by six group discussion sessions, during which participants reviewed the video-recorded interviews and provided feedback for skill enhancement. Interview I was conducted before any training, these pre-training scores serving as baseline. Training was provided alternately to groups 1 and 2. Interview II was conducted after group 1 received training and group 2 did not; group 2 served as control. Hence, interview II scores were post-training scores for group 1 and pre-training scores for group 2. Interview III was conducted after group 2 was given the same training as group I. Because of the small sample size, the Wilcoxon signed-rank test was used to examine differences between interview II and interview I (II–I) and interview III and interview II (III–II) separately.

**Fig. 1:**
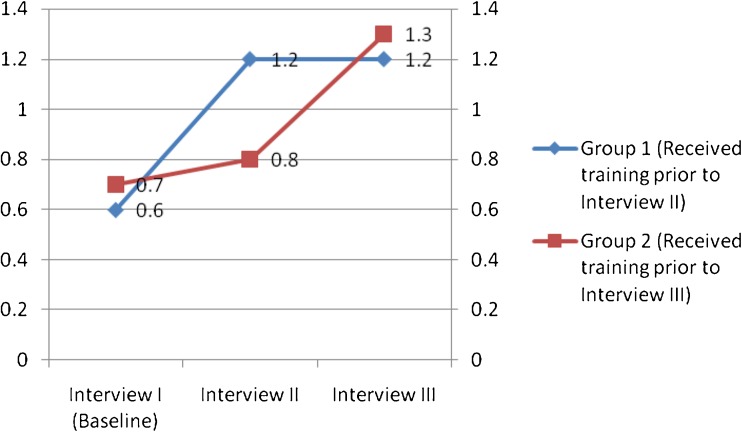
Changes in average overall scores for groups 1 and 2 in three interviews

Group 1 had a statistically significant difference in scores for all skills in interviews II and I (II–I), except for stating concrete effects (mean ± SE = 0.4 ± 0.2; P = 0.2). Group 2 had a statistically significant difference in scores only for using door openers (mean ± SE = 0.3 ± 0.1; *P* = 0.02). Thus, group 1, after undergoing training, showed marked improvement in interview skills, whereas group 2’s performance without the intervention remained the same. Group 2 had a statistically significant difference in scores for all skills in interviews III and II (III–II), whereas no such difference was observed in group 1, except for using door openers (mean ± SE = −0.3 ± 0.1; *P* = 0.04; the negative value indicates regression in competence). Figure 1 shows changes in average overall scores. Participants also reported greater self-confidence in interviewing patients and using each interview skill after the study than before, suggesting that the intervention boosted self-perceived interview competence, leading to positive changes in self-confidence.

This investigation is the first on the effectiveness of role play combined with didactic lecture, video review, and group discussion as an interview training method for new psychiatric nurses. It involved a cross-over design with a controlled intervention and compared pre- and post-training scores in three role play interview sessions. The experimental groups had significantly better post-training than pre-training scores, indicating the effectiveness of the combined training approach, which can improve the interview skills of new psychiatric nurses and thus should be adopted in pre-placement training.

Convenient to perform and interesting to participants, this approach does not incur large expenses. Participants seemed at ease during training and enjoyed the process, reporting greater self-confidence in interview proficiency and competence. Training equipped them with interview skills to deal with difficult situations encountered in everyday practice. Given the limited clinical time and reduced budgets for teaching critical interview skills to new psychiatric nurses, the proposed approach is both ideal and imperative before nurses start working in the ward.
